# Biosynthetic pathway of indole-3-acetic acid in ectomycorrhizal fungi collected from northern Thailand

**DOI:** 10.1371/journal.pone.0227478

**Published:** 2020-01-03

**Authors:** Jaturong Kumla, Nakarin Suwannarach, Kenji Matsui, Saisamorn Lumyong

**Affiliations:** 1 Department of Biology, Faculty of Science, Chiang Mai University, Chiang Mai, Thailand; 2 Center of Excellence in Microbial Diversity and Sustainable Utilization, Chiang Mai University, Chiang Mai Thailand; 3 Graduate School of Sciences and Technology for Innovation, Faculty of Agriculture, Yamaguchi University, Yamaguchi, 7 Japan; 4 Academy of Science, The Royal Society of Thailand, Bangkok, Thailand; Fujian Agriculture and Forestry University, CHINA

## Abstract

Indole-3-acetic acid (IAA) is an imperative phytohormone for plant growth and development. Ectomycorrhizal fungi (ECM) are able to produce IAA. However, only a few studies on IAA biosynthesis pathways in ECM fungi have been reported. This study aimed to investigate the IAA biosynthesis pathway of six ECM cultures including *Astraeus odoratus*, *Gyrodon suthepensis*, *Phlebopus portentosus*, *Pisolithus albus*, *Pisolithus orientalis* and *Scleroderma suthepense*. The results showed that all ECM fungi produced IAA in liquid medium that had been supplemented with L-tryptophan. Notably, fungal IAA levels vary for different fungal species. The detection of indole-3-lactic acid and indole-3-ethanol in the crude culture extracts of all ECM fungi indicated an enzymatic reduction of indole-3-pyruvic acid and indole-3-acetaldehyde, respectively in the IAA biosynthesis via the indole-3-pyruvic acid pathway. Moreover, the tryptophan aminotransferase activity confirmed that all ECM fungi synthesize IAA through the indole-3-pyruvic acid pathway. Additionally, the elongation of rice and oat coleoptiles was stimulated by crude culture extract. This is the first report of the biosynthesis pathway of IAA in the tested ECM fungi.

## Introduction

The most active phytohormone in the auxin class is indole-3-acetic acid (IAA), which is widely produced in plants and plays an important role in plant physiology including cell division and elongation, tissue differentiation, root initiation and phototropic response [1, 2]. Plants and microorganisms (bacteria and fungi) have been reported as IAA producers [3–6]. Recently, the role of microbial IAA in plant-microbe interaction including ectomycorrhizal, endophytic, pathogenic, phyllosphere and rhizospheric microbes has gained increasing amounts of attention from researchers [6–9]. L-tryptophan (L-Trp) is the main precursor for IAA synthesis by microorganisms [3, 5]. The intermediate indole compounds are a major component for the identification of the differential IAA biosynthetic pathway in microorganisms, while at least five different pathways have been described including the indole-3-acetamide (IAM), indole-3-acetonitrile (IAN), indole-3-pyruvic acid (IPyA), tryptamine (TAM) and tryptophan side-chain oxidase (TSO) pathways [3, 10]. Notably, most bacteria produce IAA via the IAM, IAN and IPyA pathways (Duca et al. 2014). IAA biosynthesis by the TSO pathway has only occurred in the bacteria *Pseudomonas fluorescens* strain CHA0 [11]. The IAM and IPyA pathways are predominant pathways for IAA biosynthesis in fungi and yeast [3, 5, 7, 12–16].

Ectomycorrhizal (ECM) associations make up a mutualistic relationship between fungi and the roots of plants by which the fungal partner receives carbohydrates through the plant’s process of photosynthesis and, in turn, provides the plant with soil nutrients and water [17]. Moreover, ECM fungi displayed the ability to increase plant tolerance under certain environmental stresses, e.g. soil pollution or drought, while also protecting the plant against root pathogens and increasing the photosynthetic rate [17–19]. Morphological and physiological changes occurred among root plants under ECM association, e.g. stimulation of lateral root development or suppression of root hair formation that were reported from a highly regulated process [20, 21]. Nevertheless, the mechanisms of ECM fungi that play a role in the root morphological and physiological changes are not yet fully understood. One possible hypothesis has proposed that phytohormones (especially IAA) or phytohormone-like substances are produced by ECM fungi in the establishment and functioning of mycorrhizal development [9, 22–26]. Gay et al. [25] and Bartel [26] suggested that IAA production in ECM association likely occurs because ECM fungi produce IAA from Trp that is present in root exudates and releases it under a symbiotic association. The IAA production ability of ECM fungi was investigated and has revealed that some pure cultures of ECM fungi, e.g. *Amanita muscaria*, *Cenococcum graniforme*, *Hebeloma hiemale*, *Paxillus involutus*, *Pisolithus tinctorius*, *Rhizopogon luteolus*, *Scleroderma sinnamariense*, *Suillus luteus*, *Su*. *bovinus*, *Tricholoma vaccinum*, *Tuber borchii* and *Tu*. *melanosporum* could produce IAA [9, 24, 27–29]. However, only the IAA biosynthesis pathway in *Tr*. *vaccinum* has been investigated [9]. Therefore, there is still a need to conduct further research on the IAA biosynthesis pathway in ECM fungi. Our previous study found that pure cultures of *Astraeus odoratus*, *Phlebopus portentosus*, and *Pi*. *albus* could produce IAA [29], but their IAA biosynthetic pathway has not yet been fully investigated. Therefore, this study aimed to investigate the IAA biosynthesis pathway of six ECM cultures including *Gyrodon suthepensis*, *Pi*. *orientalis*, *Scleroderma suthepense*, and three cultures from our previous study (*A*. *odoratus*, *Ph*. *portentosus* and *Pi*. *albus*).

## Materials and methods

### Fungal culture

Pure cultures of six ECM fungi, *A*. *odoratus* CMU53-110-7, *G*. *suthepensis* CMU55-GY1, *Ph*. *portentosus* CMU51-210-2, *Pi*. *albus* CMU53-220-2, *Pi*. *orientalis* CMU53-6 and *Sc*. *suthepense* CMU54-SC2 were from the Culture Collection of the Sustainable Development of Biological Resources Laboratory, Faculty of Science, Chiang Mai University, Chiang Mai, Thailand, and used in this study. All pure cultures were isolated from fruiting bodies that collected in northern Thailand during 2008–2014 (Table 1). Fungi were grown on modified Melin-Norkans (MMN) agar [29] and incubated at 30°C in darkness.

**Table 1:** Original, ITS GenBank accession number, collection date and host plant of fungi used in this study.

Fungal taxa	Isolate	ITS Genbank number	Location	Collection date	Associated plant	Reference
*Astraeus odoratus*	CMU53-110-7	HQ687219	Wiang Pa Pao, Chiang Rai	23 June 2010	*Dipterocapus* sp.	Kumla et al. [29]
*Gyrodon suthepensis*	CMU55-GY1	MK168570	Muang, Chiang Mai	15 June 2012	*Betula alnoides*	This study
*Phlebopus portentosus*	CMU51-210-2	HQ687224	Phrao, Chiang Mai	7 May 2008	*Mangifera indica*	Kumla et al. [29]
*Pisolithus albus*	CMU53-220-2	HQ687220	Muang, Chiang Mai	15 June 2010	*Eucalyptus camaldulensis*	Kumla et al. [29]
*Pisolithus orientalis*	CMU53-P6	JQ365188	Muang, Chiang Mai	20 June 2014	*Pinus kesiya*	This study
*Scleroderma suthepense*	CMU54-SC2	MK184287	Muang, Chiang Mai	5 May 2012	*Betula alnoides*	This study

### Culture condition for fungal IAA and related indole compounds production

All ECM fungi were grown in 25 mL of basal liquid medium (1.5 g (NH_4_)H_2_PO_4_, 0.3 g MgSO_4_·7H_2_O, 0.05 g KH_2_PO_4_, 0.02 g CaCl_2_, 0.01 g NaCl, 0.01 g FeCl_3_·6H_2_O, 0.001 g thiamine HCl, and 5.0 g glucose per one liter of distilled water, pH 6.0) supplemented with 2.0 mg/mL of L-Trp (Sigma-Aldrich, Germany, Cat No. T-0254) in 125 mL Erlenmeyer flask. Five mycelial plugs (5 mm in diameter) obtained from the periphery of the colony on MMN agar after incubation at 30°C for two weeks were transferred to each flask. Cultivation was performed in the dark at 30°C with shaking at 150 rpm on a reciprocal shaker. The culture supernatant from each flask was harvested by centrifugation at 10000 rpm for 15 min. The fungal IAA and related indole compounds in culture supernatant were extracted as shown below and investigated in every five days during incubation for 30 days. Three replications were made.

### Colorimetric assay for indole compound production

The fungal indole compound production was determined using colorimetric assay according to the method described by Tsavkelova et al. [30]. One mL of the culture supernatant was mixed with 2 mL of Salkowski’s reagent[31]. The mixture was incubated in the dark for 30 min. A positive for indole compound production was indicated by a pink to red color in the solution.

### Extraction of fungal indole metabolites

The final pH of the fungal culture supernatants was adjusted to 4.0 using 5 mol/L HCl. The fungal indole metabolite was extracted using ethyl acetate and the fungal culture supernatant obtained after pH adjustment in ratios of 2:1 (v/v) according to the method described by Chung et al. [7]. The ethyl acetate fraction was evaporated using a rotary evaporator (BUCHI Rotaryvapor model R-210/215, Switzerland). The crude culture extracts were subsequently re-dissolved in 5 mL methanol for further analyses and were stored at -20°C.

### Detection and quantification of IAA and related indole compounds

#### Thin layer chromatography

The crude ethyl acetate extract of the fungal indole metabolites was applied to thin layer chromatography (TLC) using silica gel G F254 aluminum (thickness 0.25 mm) plates (Merk, Germany). The plates were developed in a solvent mixture of *n*-hexane:ethyl acetate:isopropanol:acetic acid (40:20:5:1, v/v/v/v) according to the method described by Numponsak et al. [32]. R_f_ values were individually determined for the standards of L-Trp, IAA (Sigma-Aldrich, Germany, Cat No. I-2886,), IAM (Wako Pure Chemicals, Japan, Cat No. 325–42383), indole-3-lactic acid (ILA; Wako Pure Chemicals, Japan, Cat No. 325–45301), IAN (Sigma-Aldrich, Germany, Cat No. 129453), indole-3-ethanol (IOL; Wako Pure Chemicals, Japan, Cat No. 350–15601,), IPyA (Sigma-Aldrich, Germany, Cat No. I-17017) and TAM (Sigma-Aldrich, Germany, Cat No. 193747) under UV light (254 nm). Identification of indole compounds was also performed using chromogenic reagents by spraying with Ehmann’s [33], Ehrlich’s [33] and Salkowski’s reagents on TLC plate and subsequent heating to 90°C.

#### High performance liquid chromatography

IAA and related indole compound produced by fungal ECM culture were quantified using high performance liquid chromatography (HPLC) according to the method of Numponsak et al. [32]. HPLC analysis was performed on a Shimadzu Prominence UFLC system, coupled to a LC-20 AD pump, using a SIL-20ACHT autosampler, CTO-20 AC column oven, CBM-20A system controller and SPD-20A photodiode array detector (Shimadzu, Japan). The sample was separated on a Mightysil RP-18 (250 × 4.6 mm, 5 μm) column at 40°C. The mobile phase involved a solution of 2.5% acetic acid in deionzed water, pH 3.8 (adjusted by 10 M KOH) (A) and 80% acetonitrile in deionzed water (B). The following gradient program was used: 0–25 min, 0–20% B; 25–31 min, increased to 50% B; 31–33 min, increased to 100% B. The flow rate was 0.5 mL/min and the detection was performed with absorption at 280 and 350 nm. The injection volume was 10 μL. The presences of fungal IAA and related indole compounds were identified by comparing both the retention time and absorption spectrum with those of L-Trp, IAA, IAM, IAN, ILA, IOL, IPyA and TAM standards. The fungal IAA and related indole compounds were quantified with the calibration curve constructed with each standard. All the analyses were carried out in triplicate.

### Tryptophan aminotransferase activity

All ECM fungi were grown in 25 mL of basal liquid medium supplemented with 2.0 mg/mL of L-Trp in 125 mL Erlenmeyer flasks. Cultivation was performed at 30°C in darkness under shaking conditions at 120 rpm for one week. Fungal mycelia were harvested by filtration, followed by being washed twice with sodium phosphate buffer (0.2 mol/L, pH 7.2) and then being re-suspended in 5 mL of the same buffer and lysed by sonication with an Ultrasonic disruptor UD-201 (Tomy Seiko, Japan) at 4°C. After sonication, lysates were centrifuged at 10,000 rpm for 10 min. The clear supernatant was collected and used as a source of crude enzyme extract. The tryptophan aminotransferase activity was determined by modifying the procedure of Nutaratat et al. [16]. Enzyme activity was carried out using a mixture of 0.5 mL crude enzyme extract, and 2 mL sodium phosphate buffer containing of 50 μmol L-Trp, 20 μmol of α-ketoglutarate and 0.1 μmol pyridoxal-5-phosphate. The mixture was incubated at 30°C for 10 min. The reaction was stopped by the addition of 1 mL of 10% trichloroacetic acid. The supernatant was then collected by centrifugation. HPLC technique was used to analyze the IPyA formation. Each treatment was performed in three replications.

### Biological activities of fungal IAA

Elongation of coleoptile sections of oat (*Avena fatua* L.) and rice (*Oryza sativa* L.) was determined for the biological activity of crude fungal culture extract that was produced by ECM cultures according to the methods of previous studies [29, 34]. Oat and rice seeds were surface disinfested in a solution mixture of 0.5% sodium hypochlorite and 0.1% Tween 80 for 2 min, followed by being rinsed three times with sterile water. They were then placed on 1.0% (w/v) water agar (10 g agar per one liter of distilled water, pH 6.0). After 3 days at 25°C in darkness, 1.5−2.0 mm tips of the coleoptiles were cut, and the lengths of rice and oat coleoptiles were adjusted to 5 and 10 mm, respectively. Coleoptile sections were floated in distilled water for 2 h before being used. Crude fungal culture extract solution (containing 20 μg/mL of IAA) was prepared by dissolving in 0.5 mL of 0.1 mol/L NaOH. They were then diluted with sterile distilled water and used in this experiment. IAA solution at 20 μg/mL was used as positive control, while sterile distilled water was used as negative control. Ten coleoptile segments were used in each treatment. Coleoptile segments were floated in 12 multiple well-plates containing 5 mL of each solution and were then incubated in the dark at 25°C. The length of the coleoptile segments was measured after a 48-h floating period. Five replications were performed for each treatment.

### Statistical analysis

Statistical analyses were carried out by one-way analysis of variance (ANOVA) using SPSS program version 16.0 for Windows. Tukey’s test was used to determine any significant differences (*P* < 0.05) between the mean values of each treatment.

## Results

### Identification and quantification of IAA and related indole compounds

The first method that was employed to assess the ability of the ECM species to produce IAA in the cultures was the colorimetric assay using Salkowski’s reagent. The results indicated that all ECM fungi were positive for IAA production, as was indicated by the formation of a pink to red color by a reaction with Salkowski’s reagent. A negative IAA reaction was found in the uncultivated liquid medium.

**Fig 1:**
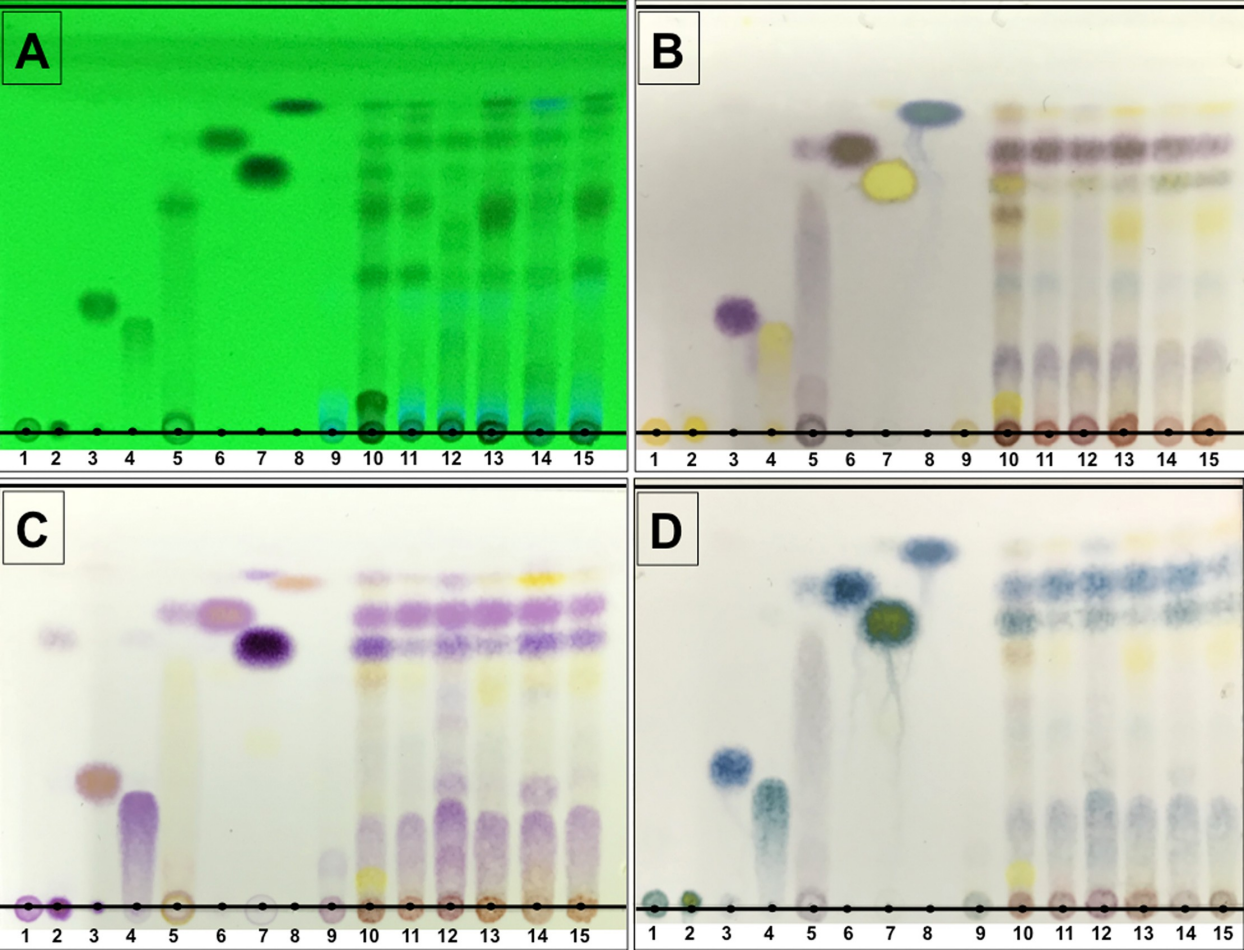
Identification of indole compounds produced by ectomycorrhizal fungi using thin layer chromatography technique under UV light (A), and chromogenic reaction reagents; Salkowski (B), Ehrlich (C) and Ehmann (D). Lane 1 = L-tryptophan (L-Trp), Lane 2 = tryptamine (TAM), Lane 3 = indole-3-acetamide (IAM), Lane 4 = indole-3-lactic acid (ILA), Lane 5 = indole-3-pyruvic acid (IPyA), Lane 6 = indole-3-acetic acid (IAA), Lane 7 = indole-3-ethanol (IOL), Lane 8 = indole-3-acetonitrile (IAN), Lane 9 = uncultivated liquid medium, Lane 10 = crude culture extract of *Astraeus odoratus*, Lane 11 = crude culture extract of *Gyrodon suthepensis*, Lane 12 = crude culture extract of *Phlebopus portentosus*, Lane 13 = crude culture extract of *Pisolithus albus*, Lane 14 = crude culture extract of *Pisolithus orientalis* and Lane 15 = crude culture extract of *Scleroderma suthepense*.

The TLC method and staining with chromogenic reagents was used to verify the presence of IAA and related indole compounds. The different R_f_ values obtained from each crude sample and the indole compound standard are presented in Fig 1 and Table 2. Separations of L-Trp and TAM were not found (R_f_ = 0). The instability of IPyA was found, as was indicated by the corresponding bands to IAA and other unknown compounds (Fig 1). The TLC chromatogram of all crude culture extract revealed the same R_f_ values of the ILA (0.22), IAA (0.68) and IOL (0.58) standards under UV light and by spraying the chromogenic reagents.

**Table 2:** Identification of indole compounds extracted from a culture of ectomycorrhizal fungi using chromogenic reagents after TLC separation.

Sample	Lane No.	R_f_ value	UV	Chromogenic reagent		
				Salkowski	Ehrlich	Ehmann
L-Trp	1	0.00	+	Yellow	Violet	Blue green
TAM	2	0.00	+	Yellow	Violet	Green
IAM	3	0.27	+	Violet	Violet pink	Blue
ILA	4	0.22	+	Yellow	Violet	Blue
IPyA	5	0.46	+	–	–	–
IAA	6	0.68	+	Violet red	Violet red	Blue
IOL	7	0.58	+	Yellow	Violet blue	Blue green
IAN	8	0.80	+	Blue green	Orange pink	Blue
Crude unculture medium extract	9	0.00	+	Yellow	Violet	Light blue green
Crude culture extract of *Astraeus odoratus*	10	0.22	+	Light yellow	Violet	Blue
		0.68	+	Violet red	Violet red	Blue
		0.58	+	Yellow	Violet blue	Blue green
Crude culture extract of *Gyrodon suthepensis*	11	0.22	+	Light yellow	Violet	Blue
		0.68	+	Violet red	Violet red	Blue
		0.58	+	Yellow	Violet blue	Blue green
Crude culture extract of *Phlebopus portentosus*	12	0.22	+	Yellow	Violet	Blue
		0.68	+	Violet red	Violet red	Blue
		0.58	+	Yellow	Violet blue	Blue green
Crude culture extract of *Pisolithus albus*	13	0.22	+	Light yellow	Violet	Blue
		0.68	+	Violet red	Violet red	Blue
		0.58	+	Yellow	Violet blue	Blue green
Crude culture extract of *Pisolithus orientalis*	14	0.22	+	Light yellow	Violet	Blue
		0.68	+	Violet red	Violet red	Blue
		0.58	+	Yellow	Violet blue	Blue green
Crude culture extract of *Scleroderma suthepense*	15	0.22	+	Light yellow	Violet	Blue
		0.68	+	Violet red	Violet red	Blue
		0.58	+	Yellow	Violet blue	Blue green

“–” = no reaction.

**Fig 2:**
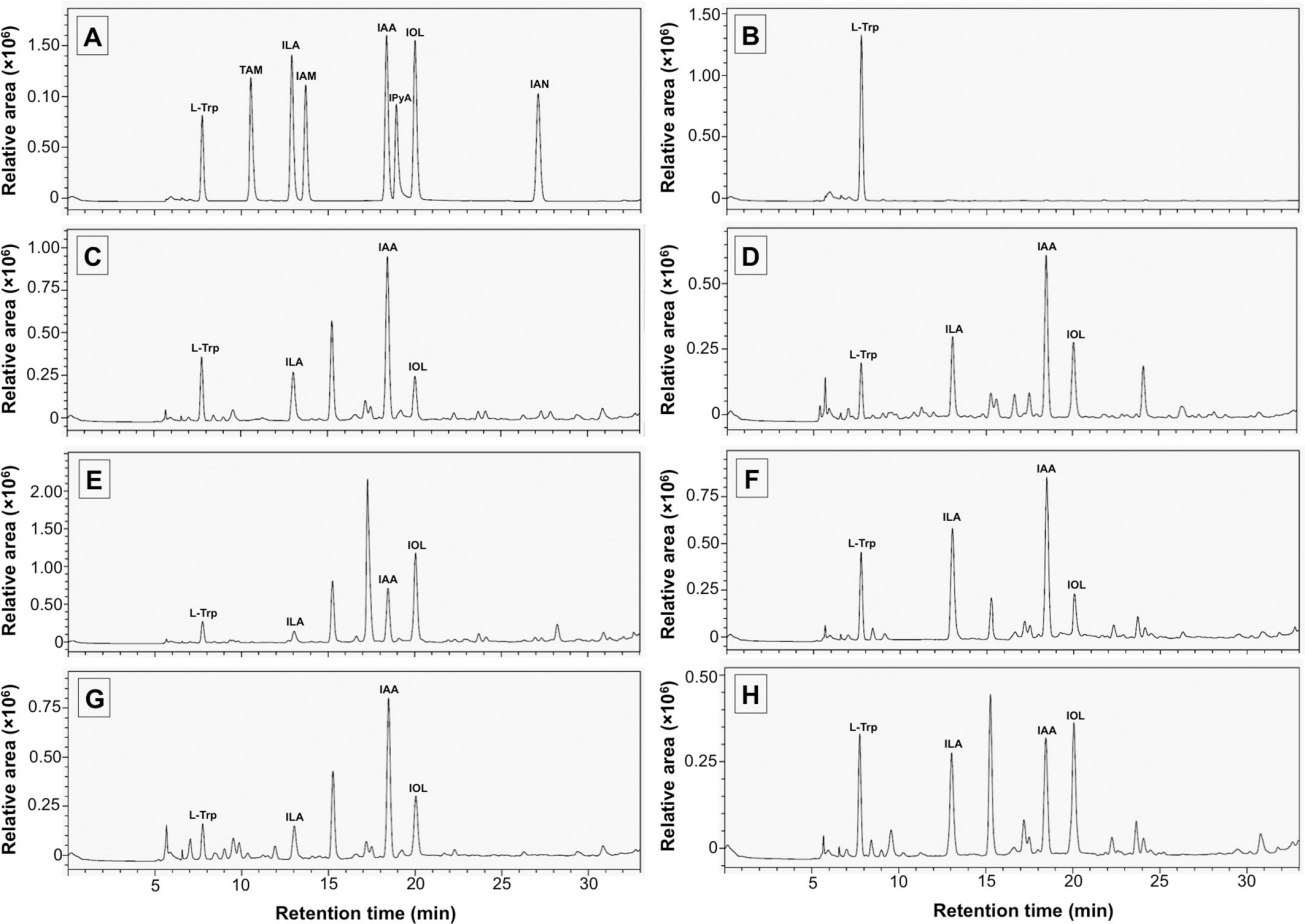
Identification of indole compounds produced by ectomycorrhizal fungi using high performance liquid chromatography technique. A. Indole compounds standard, B. Uncultivated liquid medium. C. Crude culture extract of *Astraeus odoratus*. D. Crude culture extract of *Gyrodon suthepensis*. E. crude culture extract of *Phlebopus portentosus*. F. Crude culture extract of *Pisolithus albus*. G. Crude culture extract of *Pisolithus orientalis*. H. crude culture extract of *Scleroderma suthepense*. L-Trp = L-tryptophan, TAM = tryptamine, IAM = indole-3-acetamide, ILA = indole-3-lactic acid, IPyA = indole-3-pyruvic acid, IAA = indole-3-acetic acid, IOL = indole-3-ethanol and IAN = indole-3-acetonitrile. The analyses were performed in triplicate.

The HPLC analysis was used to identify and quantify fungal IAA and other related indole compounds. The retention times of L-Trp, TAM, ILA, IAM, IAA, IPyA, IOL, and IAN were found at 7.8, 10.6, 13.1, 13.9, 18.6, 19.2, 20.2 and 27.3 min, respectively (Fig 2A). No correspondence of the IAA and other indole compounds was presented in the uncultivated medium extract (Fig 2B). The results showed that all crude fungal culture extracts presented L-Trp, ILA, IAA and IOL that corresponded to each standard (Fig 2C–2G). The identification of fungal IAA, ILA and IOL was confirmed by a co-injection with each standard.

**Fig 3:**
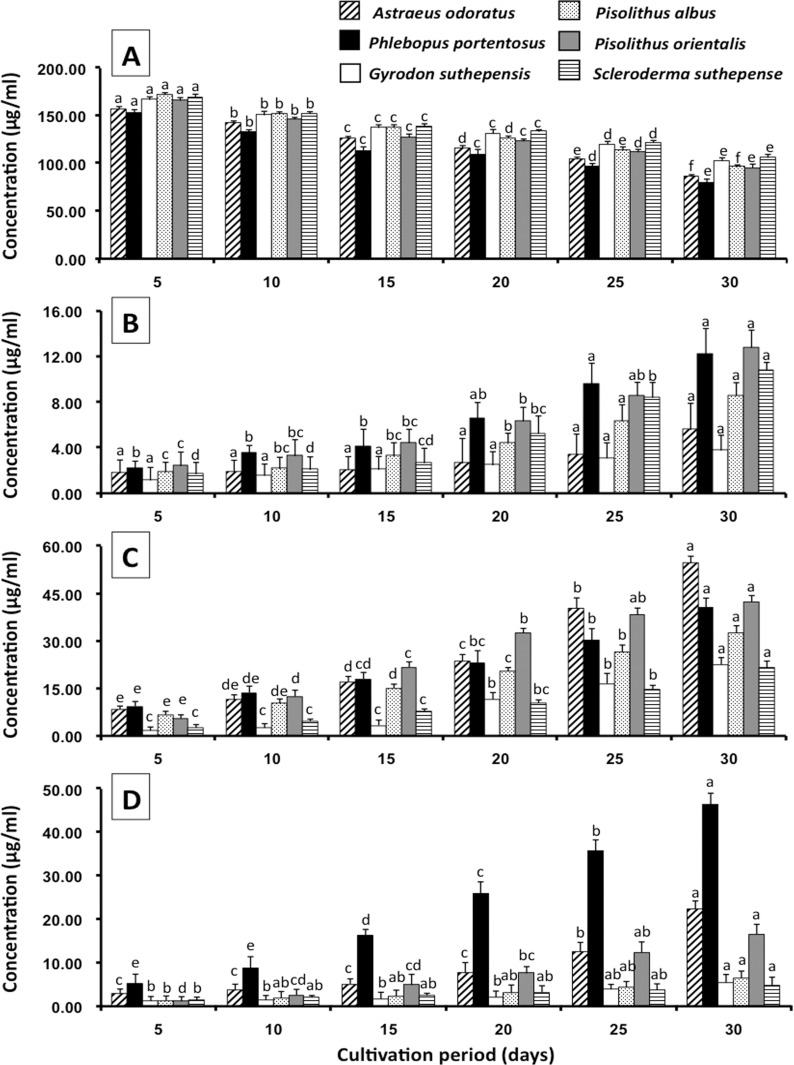
Indole compounds produced by ectomycorrhizal fungi in different cultivation periods. A. L- tryptophan, B. Indole-3-lactic acid. C. Indole-3-acetic acid. D. Indole-3-ethanol. The results are means of three replicates ± SD. Different letters above each graph indicate the significant difference (*P* < 0.05).

Under the relevant HPLC conditions, indole-3-acetaldehyde (IAAld) and IPyA were not detected, but ILA and IOL were found, as was indicated by the products obtained from the enzymatic reductions of IPyA and IAAld, respectively, in the IPyA pathway of IAA biosynthesis. Generally, the instability and accumulation of IPyA and IAAld in microbial cultures were found [3, 7, 35]. This result suggested that all fungal cultures produced IAA via the IPyA pathway. The levels of L-Trp, ILA, IAA and IOL during the cultivation period were also quantified and are presented in Fig 3 and S1 Table. The results showed that ILA, IAA and IOL levels in all crude fungal culture ethyl acetate extracts had increased when the cultivation period was increased, while the L-Trp level was found to have decreased. After 30 days of cultivation, the highest IAA level was observed in *A*. *odoratus* (54.56±2.21 μg/mL), followed by *Pi*. *orientalis* (42.27±3.18 μg/mL), *Ph*. *portentosus* (40.72±2.87 μg/mL), *Pi*. *albus* (32.65±3.25 μg/mL) and *G*. *suthepensis* (22.56±2.32 μg/mL). Conversely, the lowest IAA level was observed in *Sc*. *suthepense* (21.54±2.67 μg/mL).

### Tryptophan aminotransferase activity

**Fig 4:**
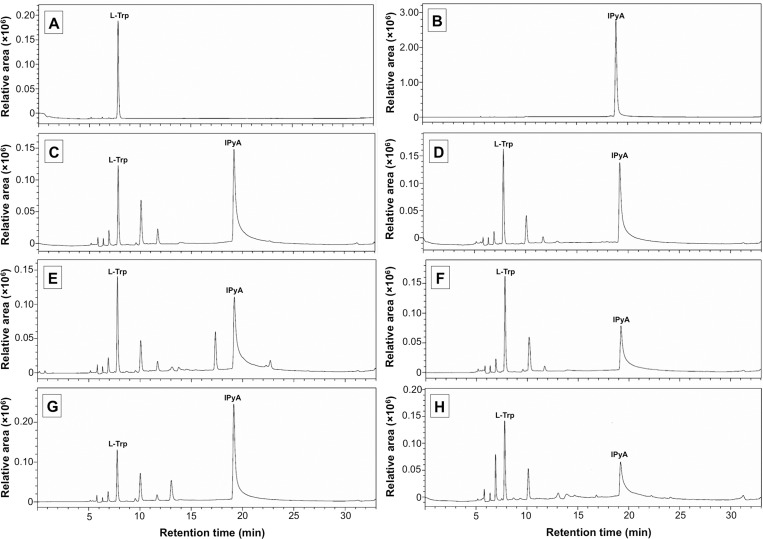
Tryptophan aminotransferase activity of crude enzyme extract of ectomycorrhizal fungi and high performance liquidchromatography detection. A. Buffer containing of L-tryptophan (L-Trp). B. Indole-3-pyruvic acid (IPyA). C. Reaction mixture of L-Trp and enzyme extract of *Astraeus odoratus*. D. Reaction mixture of L-Trp and enzyme extract of *Gyrodon suthepensis*. E. Reaction mixture of L-Trp and enzyme extract of *Phlebopus portentosus*. F. Reaction mixture of L-Trp and enzyme extract of *Pisolithus albus*. G. Reaction mixture of L-Trp and enzyme extract of *Pisolithus orientalis*. H. Reaction mixture of L-Trp and enzyme extract of *Scleroderma suthepense*. L-Trp = L-tryptophan and IPyA = indole-3-pyruvic acid. The analyses were performed in triplicate.

**Fig 5:**
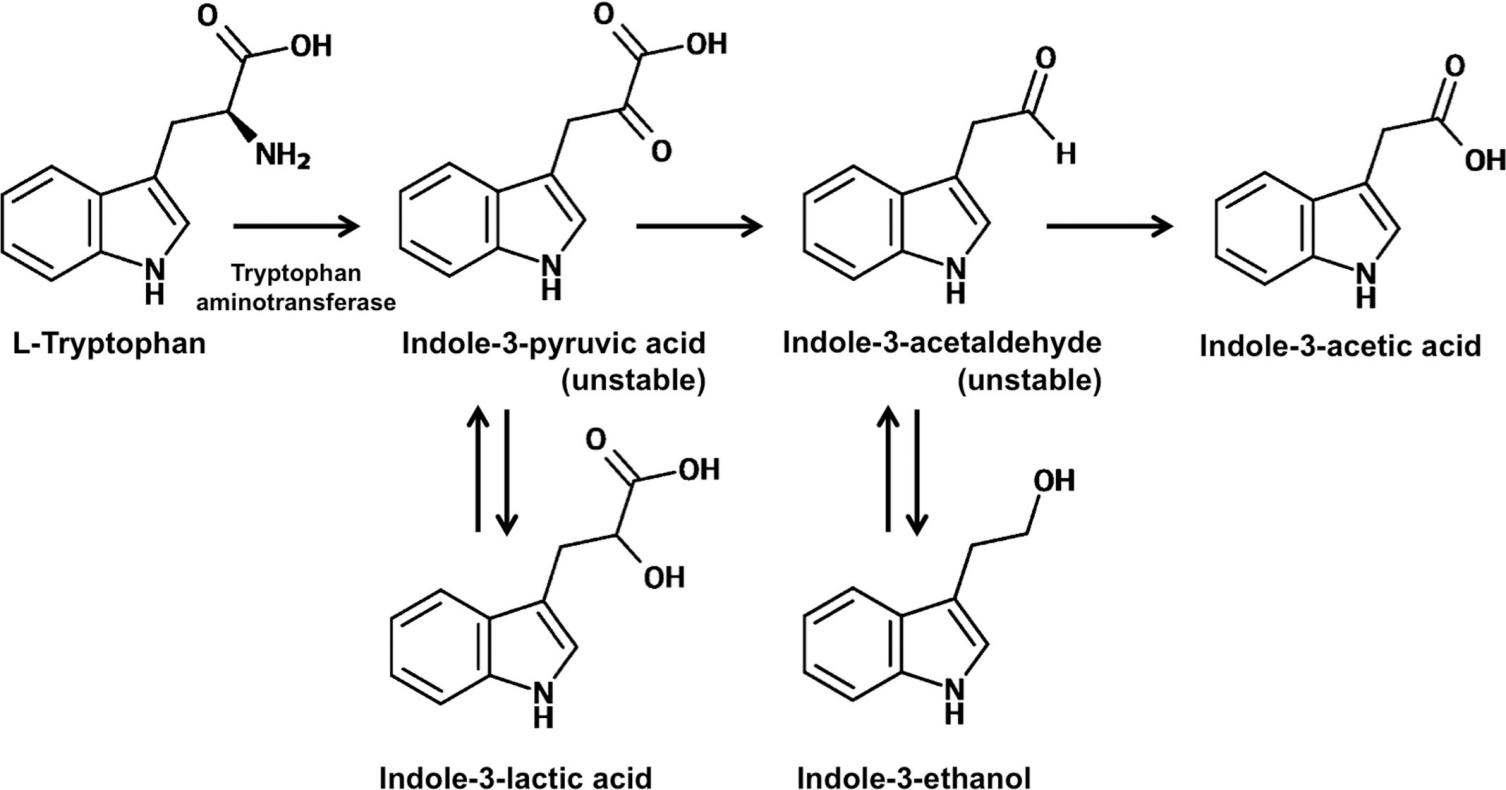
Proposed indole-3-acetic acid biosynthetic pathway of *A*. *odoratus*, *G*. *suthepensis*, *Ph*. *Portentosus*, *Pi*. *orientalis Pi*. *albus* and *Sc*. *suthepense*. The proposed pathway is based on experimental evidence observed in this study and the evidence of the intermediate compounds.

Confirmation of the IAA biosynthetic pathway by IPyA in all ECM fungi was determined by the tryptophan aminotransferase activity. The detection of IPyA in the enzyme reaction mixture using HPLC indicated the tryptophan aminotransferase activity. HPLC analysis indicated that the enzyme reaction mixture of all ECM fungi presented a peak of IPyA at a retention time of 19.2 min, which corresponded to the IPyA standard (Fig 4), whereas no correspondence was found between the indole compounds to the standards in all crude enzyme extracts of all varieties of fungi (S1 Fig). This result indicated that the crude enzyme extract of all tested fungi has tryptophan aminotransferase activity. This result supports the IPyA routed IAA biosynthesis pathway of *A*. *odoratus*, *G*. *suthepensis*, *Ph*. *Portentosus*, *Pi*. *orientalis Pi*. *albus* and *Sc*. *suthepense* (Fig 5).

### Biological activities of fungal IAA

**Fig 6:**
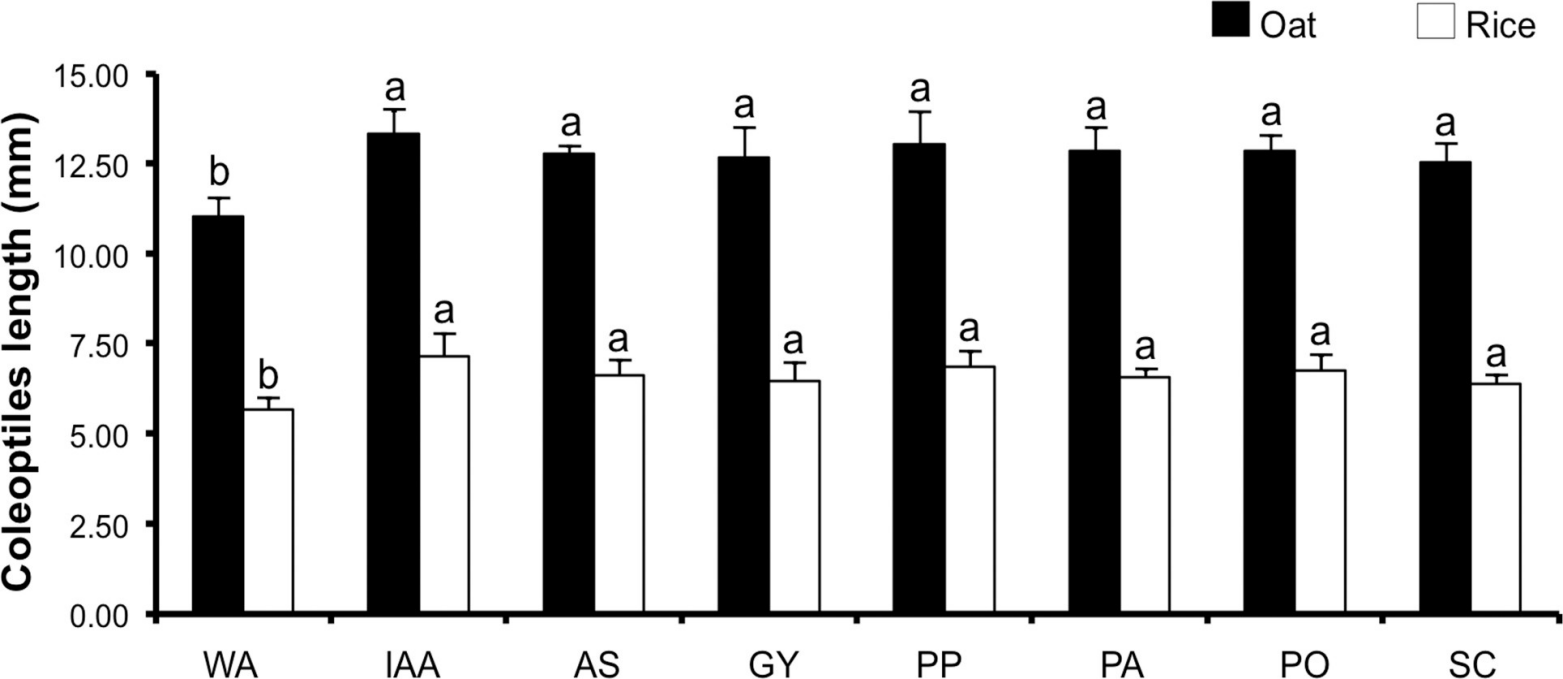
Coleoptile elongation of oat and rice. WA = distilled water treatment, IAA = IAA standard treatment, AS = crude culture extract of *Astraeus odoratus* treatment, GY = crude culture extract of *Gyrodon suthepensis* treatment, PP = crude culture extract of *Phlebopus portentosus* treatment, PA = crude IAA of *Pisolithus albus* treatment, PO = crude culture extract of *Pisolithus orientalis* treatment and SC = crude culture extract of *Scleroderma suthepense* treatment. The results are means of five replicates ± SD. Different letters above each graph indicate the significant difference (*P* < 0.05).

Coleoptile elongation assays were used to investigate the biological activity of crude fungal culture extract compared with standard IAA. The lengths of oat and rice coleoptile segments were measured after 48 h of floating in each treatment and are presented in Fig 6. It was found that the lengths of oat and rice coleoptiles that were treated with both crude fungal culture extract and IAA standard were similar, but longer than that with the distilled water treatment (control treatment).

## Discussion

IAA production by ECM cultures has been previously studied and reported by a number of researchers [9, 24, 28, 36, 37]. Our previous report (Kumla et al. 2014) and this present study found that *A*. *odoratus*, *G*. *suthepensis*, *Ph*. *portentosus*, *Pi*. *albus*, *Pi*. *orientalis* and *Sc*. *suthepense* produced IAA levels ranging from 12.84 to 54.56 μg/mL under cultivation in liquid medium that was supplemented with L-Trp. This result is similar to those of previous studies, which reported that pure cultures of ECM fungi could produce IAA after being cultured in liquid medium that had been supplemented with L-Trp. Notably, the efficiency for their IAA production varied among different fungal species and strains and was dependent upon the availability of L-Trp [9, 24, 37]. Our findings provided the first report on IAA that was produced by *G*. *suthepensis*, *Pi*. *orientalis* and *Sc*. *suthepense*.

The induction of ECM formation and the degree of efficiency during the mycobiont-plant interaction process has led to the hypothesis that phytohormone can effectively be produced by ECM fungi [23, 26, 38, 39]. This hypothesis supports the findings of Gay et al. [25] and Frankenberger and Poth [27], who showed that IAA production by *Pi*. *tictorius* and *H*. *hiemale* under mycorrhizal association affects mycorrhizal morphology in Douglas fir (*Pseudotsuga menziesii*) and pine (*Pinus halepensis*) seedlings, respectively. Rudawska and Kieliszewska-Rokicka [36] reported that the inoculation of high IAA-producing strain of *Pa*. *involutus* with Scots pine seedlings (*Pin*. *sylvestris*) induced more fine roots and significantly increased ECM formation than strains that displayed a low level of activity of IAA. Recently, Krause et al. [9] reported that the mantle sheath and the Hartig net formation increased when using the IAA overexpression strain of *Tr*. *vaccinum*. Gay et al. [25] and Niemi et al. [24] found that the conversion of L-Trp in root exudates into IAA could increase the efficiency of mycorrhizal association. Additionally, Sarjala et al. [40] reported that fungal auxin production during the initial mycorrhization state was involved in host recognition. Moreover, controlling root hair elongation during ECM development between *Pi*. *tinctorius* and eucalyptus (*Eucalyptus globulus*) seedlings was affected by the production of fungal hypaphorine (an indole alkaloid compound) and fungal IAA [41]. Splivallo et al. [28] reported IAA and ethylene production by truffles that were involved in the inhibitory activity of primary root growth and an induction in lateral root formation, respectively.

Generally, the IAA biosynthetic pathway in microorganisms was identified by the detection of indole intermediate compounds (IAM, IAN and TAM) in the culture medium, while some intermediate compounds (IPyA and IAAld) could be reversibly converted to storage compounds [3, 42]. Due to the fact that reports on the IAA biosynthetic pathway in ECM fungi are limited, there is a need to gain a clearer understanding of their IAA biosynthetic pathway. This study found that all ECM cultures produced IAA via the IPyA pathway by the presence of ILA and IOL in the fungal culture extracts, and through the course of tryptophan aminotransferase activity. The amount of IAA, ILA and IOL increased over time during the cultivation period, whereas the relative amounts of L-Trp decreased. The conversion of L-Trp to IPyA (an accumulation of ILA) could be explained by the tryptophan aminotransferase activity, along with the conversion of IPyA to IAAld (an accumulation of IOL) by IPyA decarboxylase and the conversion of IAAld to IAA by IAAld dehydrogenase. Notably, ILA and IOL have been reposted as the stable compounds and spontaneously converted from IPyA and IAAld, respectively. ILA and IOL can be readily identified in cultures as a proxy for IPyA and IAAld, respectively [3, 42]. Similarly, the study by Krause et al. [9] reported on the synthesis of IAA by the IPyA pathway in the ECM fungus, *T*. *vaccinum*. Most of the IAA production pathway that occurs in other fungi (*Neurospora crassa*, *Piriformospora indica*, *Rhizoctonia cerealis*, *R*. *solani*, *Ustilago maydis* and *U*. *esculenta*), yeast (*Rhodosporidium paludigenum*) and some bacteria (*Azospirillum brasilense*, *Bradyrhizobium elkanii*, *Gluconoacetobacter diazotrophicus*, *Pseudomonas putida* and *Rhizobium tropici*) have been observed via the IPyA pathway [13, 14, 16, 42–49].

The elongation of oat and rice coleoptile segments was stimulated by the crude fungal culture extract of all ECM fungi. Similarly, IAA production by ECM fungi (*S*. *sinnamariense* and *Suillus variegatus*) and endophytic fungi (*Colletotrichum fructicola*, *C*. *gloeosporioides*, *Tulasnella* sp. and *Muscodor cinnamomi*) could stimulate oat and rice coleoptile elongations [29, 32, 50–52]. IAA production from white rot fungus, *Pleurotus ostreatus* significantly increased the lengths of wheat coleoptile segments [53]. Moreover, Niemi et al. [24] found that IAA production by *Pa*. *involutus* could effectively improve the growth of the Oregon pine (*Pseudotsuga menziesii*) seedlings.

## Conclusions

Our results suggest that all tested ECM cultures used L-Trp as a primary source to synthesize IAA through the IPyA pathway by detection of ILA and IOL, which are intermediate indolic compounds of the IPyA pathway and of tryptophan 2-monooxygenase activity. Crude fungal culture containing IAA is known to play a role in plant-growth promotion by the stimulation of coleoptile elongation. Further studies of all ECM fungal isolates are required to fully understand the role of IAA in the development of ECM’s association with host plants and to evaluate their plant growth promoting abilities.

## Supporting information

S1 Table Indole compounds in crude culture extract of ectomycorrhizal fungi in different cultivation periods. (DOCX)

S1 Fig Crude enzyme extract from mycelia of ectomycorrhizal fungi and high performance liquid chromatography detection. A. Indole compound standards. B. Uncultivated liquid medium extract. C. Crude enzyme extract of *Astraeus odoratus*. D. Crude enzyme extract of *Gyrodon suthepensis*. E. crude enzyme extract of *Phlebopus portentosus*. F. Crude enzyme extract of *Pisolithus albus*. G. Crude enzyme extract of *Pisolithus orientalis*. H. crude enzyme extract of *Scleroderma suthepense*. L-Trp = L-tryptophan, TAM = tryptamine, IAM = indole-3-acetamide, ILA = indole-3-lactic acid, IPyA = indole-3-pyruvic acid, IAA = indole-3-acetic acid, IOL = indole-3-ethanol and IAN = indole-3-acetonitrile. The analyses were performed in triplicate.
(DOC)
