# Supplementary material for: Biosynthetic pathway of indole-3-acetic acid in ectomycorrhizal fungi collected from northern Thailand
Source: PLoS One. 2020 Jan 3;15(1):e0227478. doi: 10.1371/journal.pone.0227478 (PMC6941825; doi:10.1371/journal.pone.0227478)
Supplement: S1 Table — (DOCX) [file pone.0227478.s001.docx]

**S1 Table** Indole compounds in crude culture extract of ectomycorrhizal fungi in different cultivation periods.

| **Fungal taxa** | **Cultivation period (days)** | **Indole compounds (µg/ml)** | | | | | | | | | | | |
| --- | --- | --- | --- | --- | --- | --- | --- | --- | --- | --- | --- | --- | --- |
|  |  | **L-Trp** | | | **ILA** | | | **IAA** | | | **IOL** | | |
|  |  | R1 | R2 | R3 | R1 | R2 | R3 | R1 | R2 | R3 | R1 | R2 | R3 |
| *Astraeus odoratus* | 5 | 159.97 | 154.05 | 155.90 | 2.45 | 1.79 | 1.11 | 7.35 | 9.43 | 8.40 | 4.07 | 1.78 | 2.90 |
|  | 10 | 144.94 | 140.16 | 142.08 | 1.25 | 1.87 | 2.49 | 9.90 | 11.46 | 12.98 | 3.66 | 5.08 | 2.26 |
|  | 15 | 127.87 | 126.47 | 122.73 | 3.55 | 0.53 | 2.05 | 15.50 | 18.82 | 17.14 | 6.31 | 5.08 | 3.85 |
|  | 20 | 115.41 | 118.74 | 112.47 | 2.06 | 4.25 | 1.51 | 23.56 | 21.49 | 25.77 | 10.06 | 7.62 | 5.16 |
|  | 25 | 104.11 | 106.94 | 102.25 | 5.18 | 2.04 | 2.81 | 39.08 | 42.68 | 45.53 | 14.68 | 10.38 | 12.56 |
|  | 30 | 87.73 | 82.28 | 87.98 | 7.78 | 3.32 | 5.57 | 52.45 | 54.63 | 56.86 | 24.26 | 22.28 | 20.53 |
| *Gyrodon suthepensis* | 5 | 163.88 | 170.10 | 166.22 | 1.67 | 1.04 | 0.37 | 2.81 | 1.66 | 0.57 | 2.25 | 0.15 | 1.21 |
|  | 10 | 153.76 | 152.03 | 145.72 | 2.72 | 1.73 | 0.76 | 3.86 | 2.54 | 1.18 | 0.43 | 1.40 | 2.47 |
|  | 15 | 135.10 | 135.84 | 140.99 | 3.23 | 0.88 | 2.03 | 3.36 | 1.69 | 5.01 | 3.24 | 1.66 | 0.18 |
|  | 20 | 134.95 | 132.88 | 126.06 | 3.66 | 1.25 | 2.43 | 9.43 | 13.71 | 11.56 | 0.62 | 3.52 | 2.02 |
|  | 25 | 120.38 | 122.75 | 115.78 | 1.01 | 2.68 | 4.33 | 13.29 | 10.79 | 16.53 | 5.26 | 2.02 | 2.97 |
|  | 30 | 104.78 | 98.97 | 104.33 | 5.12 | 3.74 | 2.43 | 24.86 | 22.58 | 20.23 | 7.13 | 3.40 | 5.21 |
| *Phlebopus portentosus* | 5 | 155.66 | 148.95 | 154.13 | 2.79 | 2.11 | 1.45 | 10.78 | 9.24 | 7.68 | 3.04 | 5.32 | 7.58 |
|  | 10 | 136.90 | 131.73 | 130.36 | 2.94 | 4.10 | 3.54 | 15.74 | 13.69 | 11.58 | 12.10 | 9.71 | 7.33 |
|  | 15 | 117.56 | 108.53 | 112.47 | 4.10 | 2.61 | 5.62 | 20.07 | 15.95 | 18.02 | 19.79 | 16.89 | 18.33 |
|  | 20 | 114.72 | 109.33 | 103.89 | 8.97 | 4.07 | 6.53 | 26.95 | 23.17 | 19.34 | 25.33 | 27.96 | 30.63 |
|  | 25 | 100.02 | 96.63 | 93.48 | 9.28 | 7.62 | 10.92 | 26.81 | 30.38 | 33.93 | 38.27 | 35.77 | 33.25 |
|  | 30 | 83.56 | 79.07 | 74.54 | 13.26 | 11.02 | 8.81 | 43.59 | 40.73 | 37.85 | 50.09 | 42.77 | 46.42 |
| *Pisolithus albus* | 5 | 174.45 | 171.92 | 169.37 | 0.99 | 2.73 | 1.84 | 7.78 | 6.68 | 5.54 | 2.38 | 1.31 | 0.28 |
|  | 10 | 151.98 | 149.58 | 154.21 | 3.15 | 1.10 | 2.15 | 11.36 | 10.32 | 9.31 | 0.52 | 1.82 | 3.33 |
|  | 15 | 140.56 | 134.20 | 137.38 | 1.77 | 4.01 | 2.90 | 16.35 | 13.89 | 15.17 | 2.76 | 1.02 | 4.51 |
|  | 20 | 124.34 | 128.74 | 126.55 | 4.41 | 3.56 | 5.28 | 21.65 | 20.42 | 19.28 | 3.94 | 5.01 | 2.86 |
|  | 25 | 110.19 | 113.80 | 117.39 | 7.57 | 4.67 | 6.13 | 24.36 | 28.75 | 26.52 | 4.87 | 3.81 | 5.87 |
|  | 30 | 96.16 | 93.58 | 98.66 | 8.57 | 10.70 | 6.40 | 32.62 | 35.89 | 29.40 | 6.84 | 3.50 | 5.06 |
| *Pisolithus orientalis* | 5 | 169.15 | 165.79 | 162.80 | 1.13 | 1.42 | 3.61 | 6.76 | 4.43 | 5.24 | 2.26 | 1.06 | 0.17 |
|  | 10 | 148.45 | 143.90 | 146.17 | 4.70 | 1.77 | 3.30 | 12.41 | 14.48 | 10.45 | 1.18 | 2.56 | 3.87 |
|  | 15 | 130.82 | 127.25 | 123.63 | 4.22 | 2.03 | 6.42 | 23.45 | 21.61 | 20.18 | 2.45 | 4.92 | 7.34 |
|  | 20 | 125.41 | 123.63 | 120.66 | 6.30 | 7.56 | 5.10 | 36.05 | 32.98 | 34.45 | 6.11 | 7.60 | 9.12 |
|  | 25 | 114.90 | 111.60 | 108.42 | 9.74 | 8.55 | 7.38 | 40.63 | 37.85 | 36.28 | 9.90 | 12.38 | 14.80 |
|  | 30 | 99.67 | 94.18 | 91.34 | 15.37 | 10.26 | 12.64 | 45.48 | 42.22 | 39.12 | 14.36 | 18.81 | 16.45 |
| *Scleroderma suthepense* | 5 | 167.15 | 173.20 | 168.00 | 3.01 | 1.98 | 0.93 | 3.33 | 2,59 | 1.77 | 0.69 | 1.42 | 2.20 |
|  | 10 | 154.31 | 152.19 | 150.01 | 3.79 | 2.65 | 1.49 | 6.30 | 3.02 | 4.66 | 1.50 | 2.57 | 2.03 |
|  | 15 | 141.62 | 138.08 | 134.54 | 5.39 | 4.17 | 3.14 | 6.59 | 6.12 | 5.47 | 2.87 | 2.34 | 1.76 |
|  | 20 | 130.22 | 133.22 | 134.54 | 6.25 | 4.66 | 7.78 | 8.82 | 11.84 | 10.38 | 1.70 | 4.72 | 3.20 |
|  | 25 | 124.12 | 121.81 | 119.44 | 9.74 | 7.07 | 8.36 | 16.19 | 14.79 | 13.29 | 5.16 | 2.27 | 3.69 |
|  | 30 | 109.51 | 106.12 | 102.47 | 14.14 | 11.78 | 12.37 | 18.89 | 24.23 | 21.50 | 3.01 | 6.71 | 4.82 |

Data were from triplicate. L-Trp = L-tryptophan, ILA = indole-3-lactic, IAA = indole-3-acetic acid and IOL = indole-3-ethanol.
