# Supplementary material for: Biosynthetic pathway of indole-3-acetic acid in ectomycorrhizal fungi collected from northern Thailand
Source: PLoS One. 2020 Jan 3;15(1):e0227478. doi: 10.1371/journal.pone.0227478 (PMC6941825; doi:10.1371/journal.pone.0227478)
Supplement: S1 Fig — A. Indole compound standards. B. Uncultivated liquid medium extract. C. Crude enzyme extract of Astraeus odoratus. D. Crude enzyme extract of Gyrodon suthepensis. E. crude enzyme extract of Phlebopus portentosus. F. Crude enzyme extract of Pisolithus albus. G. Crude enzyme extract of Pisolithus orientalis. H. crude enzyme extract of Scleroderma suthepense. L-Trp = L-tryptophan, TAM = tryptamine, IAM = indole-3-acetamide, ILA = indole-3-lactic acid, IPyA = indole-3-pyruvic acid, IAA = indole-3-acetic acid, IOL = indole-3-ethanol and IAN = indole-3-acetonitrile. The analyses were performed in triplicate. (DOC) [file pone.0227478.s002.doc]

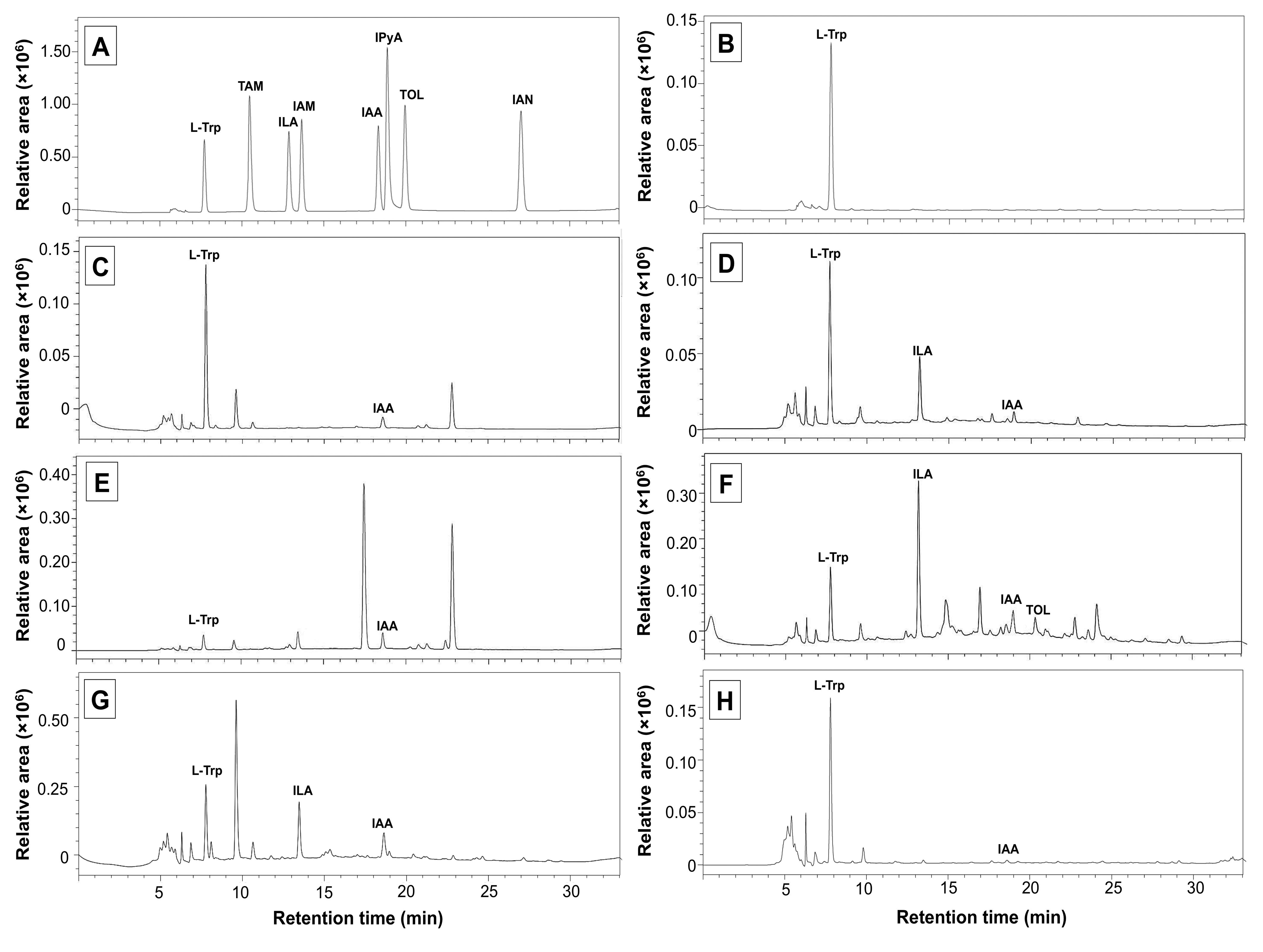


**S1 Fig.** Crude enzyme extract from mycelia of ectomycorrhizal fungi and high performance liquidchromatography detection. A. Indole compound standards. B. Uncultivated liquid medium extract. C. Crude enzyme extract of *Astraeus odoratus*. D. Crude enzyme extract of *Gyrodon suthepensis*. E. crude enzyme extract of *Phlebopus portentosus*. F. Crude enzyme extract of *Pisolithus albus*. G. Crude enzyme extract of *Pisolithus orientalis*. H. crude enzyme extract of *Scleroderma suthepense*. L-Trp = L-tryptophan, TAM = tryptamine, IAM = indole-3-acetamide, ILA = indole-3-lactic acid, IPyA = indole-3-pyruvic acid, IAA = indole-3-acetic acid, IOL = indole-3-ethanol and IAN = indole-3-acetonitrile. The analyses were performed in triplicate.
